# Correspondence: Laparoscopic repair of abdominal wall hernia - “How I do it” - synopsis of a seemingly straightforward technique

**DOI:** 10.1186/s12893-015-0080-7

**Published:** 2015-08-19

**Authors:** Christophe R. Berney

**Affiliations:** Department of Surgery, Bankstown-Lidcombe Hospital, University of New South Wales, Eldridge Road, Bankstown, NSW 2200 Australia

## Abstract

Abdominal wall hernia repairs are commonly performed worldwide in general surgery. There is still no agreed consensus on the optimal surgical approach. Since the turn of the twenty-first century, minimally invasive techniques have gained in popularity as they combine the advantages of limited abdominal wall dissection, reduced post-operative pain and risk of complications, and shorter hospital stay. Although the added cost incurred by using sophisticated laparoscopic instruments may be quite substantial, it is precisely counterbalanced by an improved morbidity rate, faster discharge home and time to return to work. Laparoscopic abdominal wall hernia repair is often challenging, as it requires good anatomical knowledge, eye-hand coordination and diversified laparoscopic skills. The objective of this article is not to present another set of personal data and to compare it with already published results on this matter, but simply to offer comprehensive guidelines on the practical aspects of this relatively new technique. Some of these steps have already been discussed but most of the time in a scattered way in the surgical literature, while others are the fruit of a personal expertise grasped over the years.

## Main text

Mesh repair of primary ventral abdominal wall and incisional hernias is a common procedure performed worldwide by general surgeons. The use of mesh-based techniques has revolutionized our practice as it has significantly dropped the unacceptably elevated long-term recurrence rates, previously reported to be as high as 60 % with a simple primary open surgical repair without mesh [1, 2]. Except from general acceptance that use of prosthetic material for good hernia repair is essential, it is extremely difficult to find agreed international consensus on what would be the most optimal surgical approach for treatment of a particular abdominal wall defect. This is in part due to the ongoing development and release of new prosthetic materials, improvement of existing technologies and description of innovative surgical techniques.

Since the turn of the twenty-first century minimally invasive laparoscopic approach has gained in popularity and may become the preferred method for hernia defects smaller than 10 cm in width [3]. The advantages of this technique over traditional open repair approach are reduced post-operative pain and wound complication rates, quicker recovery and return to normal physical activities, the potential to identify and repair any associated subclinical (occult) hernia defect concomitantly [4], including Swiss-cheese weaknesses, and improved cosmesis.

Unlike the laparoscopic technique for repair of inguinal hernias that requires a slow and often laborious learning curve to gaining complete familiarity with the anatomy of the groin [5, 6], the use of such approach to ventral or incisional hernia repairs is relatively simpler and more straightforward to acquire as the operator is not restricted by a confined operative space and the overall surgical view is generally better. Having said that it is still very important to become familiar with the anatomical landmarks, to possess good eye-hand coordination and suitable skills in minimally invasive surgical techniques in order to minimize the potential risk of developing major complications such as small bowel injury, or increase the hernia recurrence rate.

The concept of laparoscopic approach for repair of ventral and incisional hernias (LRVIH) is inspired from the principles of the open retrorectus tension-free mesh repair (sublay) technique championed by Rives-Stoppa, where the prosthesis is held in place between the muscle and its posterior sheath by transfixing sutures and intra-abdominal pressure [7, 8]. However, due to its simplicity and the fact that it requires much less extensive lateral dissection laparoscopic repair differs significantly in the sense that the mesh is preferentially secured directly in the abdominal cavity as the so-called IntraPeritoneal Onlay Mesh (IPOM) technique, first described by LeBlanc and Booth in 1993 [9]. In other words with the IPOM approach a considerable size prosthetic mesh can be easily inserted and secured to the posterior abdominal wall offering a wide overlap of the defect and greater mesh to hernia ratio, thus improving even further long-term clinical outcome (low recurrence rate) [10]. In comparison both the open onlay or sublay techniques would necessitate either raising large subcutaneous flaps or developing considerable space behind the rectus muscles bilaterally to deploy a similar large-sized mesh, thus resulting in significantly higher morbidity rate.

Apart from its relatively higher cost, other disadvantages of the laparoscopic technique compared to a standard open approach are the potential risk of visceral injury and not being able to restore the anatomy and physiology of a fully engineered abdominal wall, if the fascial defect has been left open before securing the mesh to its edges [11, 12].

Abdominal wall hernia disease is often a complex problem and the treatment options are numerous, especially with the ongoing development of new prosthetic materials or tacking devices. As such, a ‘reductionist’ scientific approach to study and conclude what would be the best treatment option according to ‘evidence-best medicine’ is unfeasible and beyond the scope of this article. The purpose of this ‘How I do it’ paper is therefore to offer an experienced surgical opinion on the practical aspects of LRVIH for those interested to learn and/or improve their technique for the benefit of their patients. Indeed, the key for a successful laparoscopic repair relies on the repetition of identical moves that follow a very detailed ‘step by step’ scheme. Some of the basic rules here have already been discussed in various formats in the surgical literature, while others are the fruit of a personal expertise grasped over the years. I will initially and mainly talk about the IPOM repair, which is the most widely used and simpler technique to learn. I will also discuss another approach, which is our preferred method for abdominal wall hernia repairs whenever suitable, namely the TransAbdominal PrePeritoneal (TAPP) technique that is more commonly used in inguinal hernia repairs, and briefly about the Totally Extraperitoneal (TEP) approach for Spigelian hernias. I do not intend to talk about laparoscopic parastomal hernia repair, which is a more complex procedure than standard LRVIH and should be only attempted by expert laparoscopic hernia surgeons. Approval to prepare this manuscript was given by our local Ethics Committee of the Department of Surgery at Bankstown-Lidcombe Hospital.

### Surgical technique

The indications for laparoscopic repair of abdominal wall hernias are essentially the same as for open surgery and absolute contraindications to this method, although quite rare would include patients with severe cardiovascular, pulmonary and liver impairment, intra-abdominal sepsis, children and pregnancy. Specific contraindications are the presence of a strangulated hernia, or patients in whom a safe access to the intra-abdominal cavity cannot be achieved due to dense adhesions. Relative contraindications include non-reducible (incarcerated), large size (>10 cm in diameter) and loss of domain hernias. This will of course depend on the surgeon’s skills.

Preoperative **s**pecific measures are essential for patients on antiplatelet medication and anticoagulant such as aspirin, clopidogrel or warfarin. If aspirin has been prescribed as secondary prevention treatment, it will be withheld for one week before surgery. Otherwise, as a primary prevention the aspirin won’t be stopped and the patient will be warned about the higher risk of post-operative bleeding. In case of dual antiplatelet regimen following previous coronary stenting, the clopidogrel will be discontinued preferentially ten days prior to the procedure and the aspirin maintained unless otherwise instructed by the cardiologist. Finally, for patients on warfarin the decision will be adapted depending on the indication for anticoagulation and in collaboration with their specialist cardiologist and/or hematologist. Whether anticoagulation can only be withheld for several hours or a few days, preference will still be for a laparoscopic IPOM approach as the level of surgical abdominal wall dissection will still be minimal as compared to a traditional open technique.

In theatre, the patient is shaved using a clipper in order to avoid skin micro traumatism, which can potentially increase the risk of infection [13]. He/she is also asked to empty his/her bladder before surgery in order to avoid urine catheterization and potential risk of catheter associated UTI. As a preference an indwelling catheter (IDC) will be only used if the surgery is likely to last for more than two hours, if a male patient suffers from prostatism, or if the hernia defect is suprapubic. Single-shot preoperative low-molecular weight heparin (Enoxaparin 20 mg) and prophylactic first generation cephalosporin (Cephalothin 1gr) injections are preferably used. The procedure is performed under general anesthesia and muscular relaxation. Sequential calf compressors are used throughout the entire length of the procedure. The patient is placed in a supine position with both arms tucked in at his side. If deemed necessary, an orogastric tube is inserted to decompress the stomach and will be removed at the end of the procedure. The surgeon and camera holder stand on the side opposite to the TV monitor, which position will vary depending on the location of the abdominal wall defect and suspected presence of underlying adhesions. As most ventral/incisional hernias arise at the level of the midline through the *linea alba*, the surgeon and his assistant should preferentially stand on the left side of the patient. Indeed, the descending colon is narrower and more dorsally situated than the ascending colon; it is therefore preferable to position the working ports from the left side of the abdomen as it represents the most lateral insertion site, meaning the further away from the hernia defect.

#### Laparoscopic IntraPeritoneal Onlay Mesh **(**IPOM) technique

**Fig. 1:**
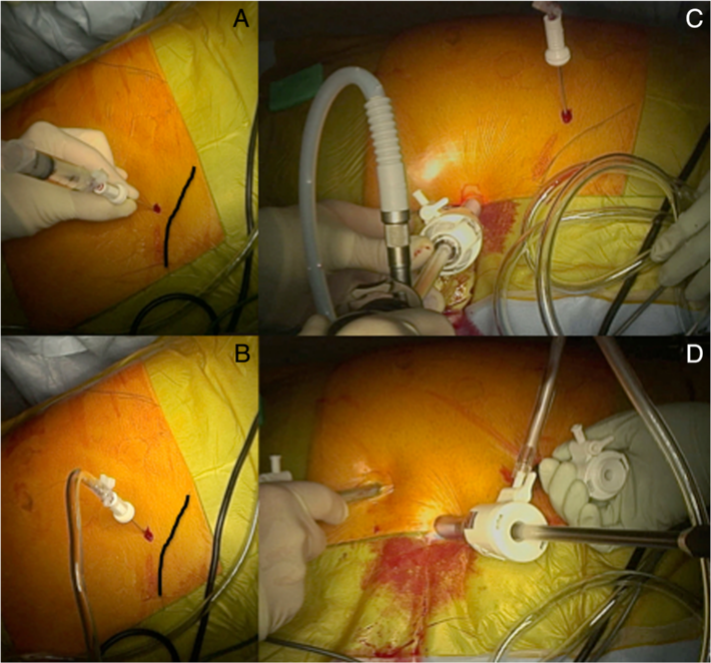
**a**) Veress needle insertion at Palmer’s point below the left costal margin; **b**) 12mmHg pneumoperitoneum; **c**) First optical trocar at level of left anterior axillary line; **d**) 5mm working ports insertion under direct vision

After skin disinfection with betadine, application of sterile drapes and covering of the abdominal cavity with an antimicrobial 3M™ Steri-Drape (Ioban™ 2, North Ryde, NSW, Australia) a pneumoperitoneum is created using a Veress needle inserted in the left upper quadrant at Palmer’s point, which is situated around 3 cm below the costal border in the mid-clavicular line. This is the safest point of entry using this method Fig. 1a, b). A single use bladeless 12 mm optical trocar is then inserted at the level of the left anterior axillary line, midway between the iliac crest and the costal margin, followed up by two blunt 5 mm working ports introduced under direct vision, either on each side of the 12 mm port laterally or both on the same side (Fig. 1c, d). A pneumoperitoneum is maintained at a maximum pressure of 12 mm Hg with CO_2_ insufflation and a 10 mm 30° telescope is used until completion of the procedure.

**Fig. 2:**
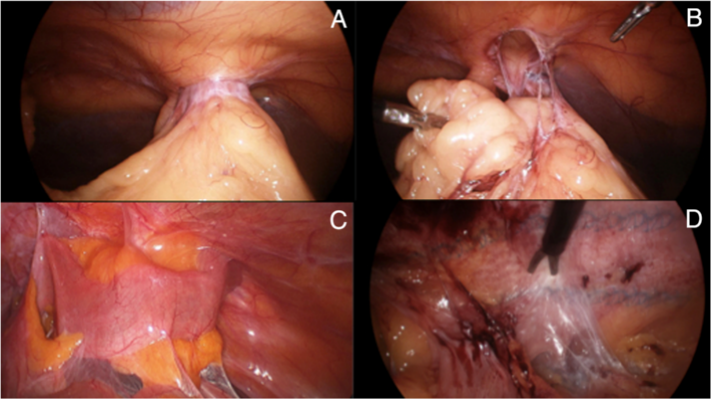
**a**) Incarcerated segment of greater omentum in ventral hernia; **b**) Reduction of hernia sac content; **c**) Dense adhesions between small bowel and abdominal wall; **d**) Sharp dissection between prosthetic material and underlying viscus

**Fig. 3:**
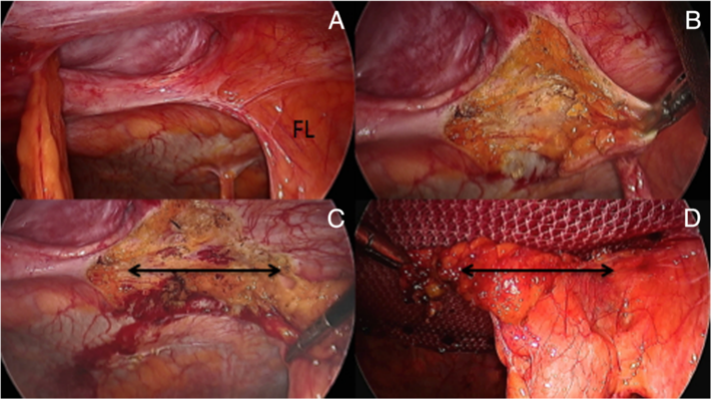
**a**) Laparoscopic view of a reduced epigastric hernia (FL: falciform ligament); **b**) Progressive dissection of the FL; **c**) FL mobilisation completed (size arrow 4cm); **d**) FL covering cephalad part of mesh

Following an overall inspection of the abdominal cavity, the first step of the procedure is to perform a careful and meticulous division of all the adhesions between the abdominal wall and underlying viscus that are likely to interfere with the hernia repair if left untouched. In other words freeing the entire abdominal wall zone where the mesh will be subsequently fixed. This also includes dissecting the adhesions that have formed within the hernia sac in order to completely reduce its content, consisting mainly of greater omentum but also loops of small bowel and/or transverse colon on some occasions (Fig. 2a, b). The best technique of adhesiolysis is by cold and sharp two-handed dissection with scissors, only utilizing an energy source such as the ultrasonic scalpel as a secondary option thus minimizing the potential and sometimes devastating risk of small bowel injury. This is even more important when the repair is for recurrent ventral hernia repair as dense adhesions can still form between the prosthetic mesh and underlying viscus (Fig. 2c, d). If the defect is extending cephalad towards the epigastrium, then the *ligamentum teres* of the liver along with part of the falciform ligament will also need to be dissected off the abdominal wall (Fig. 3). That way, optimal hernia margins around the edges of all identified defects can be obtained. This distance should be at least 3 cm in every direction, but preferably up to 5 cm as generally recommended for optimal repair [14]. The degree of mesh overlapping will vary on the size of the weakness, but also if the hernia defect has been primarily closed or not as it will significantly change the overall surface area where the prosthesis is in contact with the abdominal wall. It is therefore preferable to talk about the mesh-to-defect ratio rather than overlap [15]. The higher the ratio is, the less likely the risk of recurrence. I recommend this ratio to be at least >1 when the defect is left open.

Conventional IPOM technique corresponds to a ‘bridging’ repair where the abdominal wall defect is left open (tension-free) and covered by the mesh. In this situation post-operative seroma formation will inevitably occur as well as residual abdominal bulging at the site of hernia repair, which is not cosmetically rewarding and can be regarded by the patients as a hernia recurrence. Another important disadvantage is that no attempt is made to restore the integrity of the *linea alba*, which is essential for achieving proper anatomical and physiological function of the abdominal wall. This is the reason why, whenever achievable without creating too much tension, I recommend primarily closing the defect before performing the mesh repair. This also maximizes the amount of tissue ingrowth into the mesh as the surface area between the prosthesis and the abdominal wall is significantly increased. Such variation is called ‘augmentation repair’, or IPOM-Plus [16]. It is important to consider the shape of the abdominal wall defect prior to opting for a primary closure or not. Indeed, the more elliptical the opening the likeliest primary approximation of the hernia contours will be achievable with acceptable wound edge tension. Conversely the further the length-to-width ratio of the hernia defect approaches the value of 1, meaning the more circular it is in shape, the greatest the tension to the wound edge will apply if trying to primarily close it, and then a ‘bridging’ repair will be more appropriate. Laparoscopic bilateral component separation technique for increasing the chance of primary approximation of the rectus abdominis muscles has been recently proposed [17], but we will not expand on the subject as this is beyond the scope of this article.

**Fig. 4:**
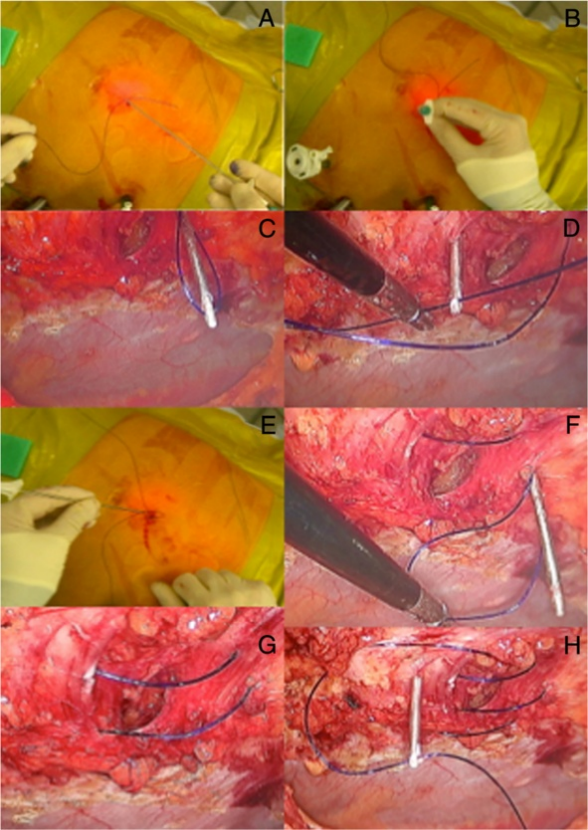
Transabdominal ‘shoelacing’ technique. **a** + **b**) Absorbable PDS suture introduced through stab wound using Endoclose device; **c**) Laparoscopic view of PDS suture; **d**) Endoclose reintroduced through same stab incision but using different passageway and grasping end of PDS suture; **e**) Loop of PDS exteriorised; **f**) Same PDS suture reintroduced through abdominal wall using another passageway; **g**) Final loose figure-of-eight obtained; **h**) Second PDS suture

**Fig. 5:**
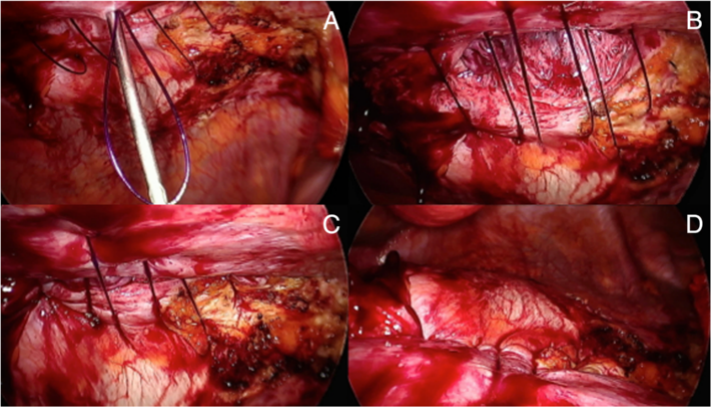
**a**) Placement of a third loose PDS suture; **b**) All three sutures adequately positioned; **c**) Sutures pulled up together maintaining edges of defect in juxtaposition; **d**) Knotted sutures and totally closed defect

**Fig. 6:**
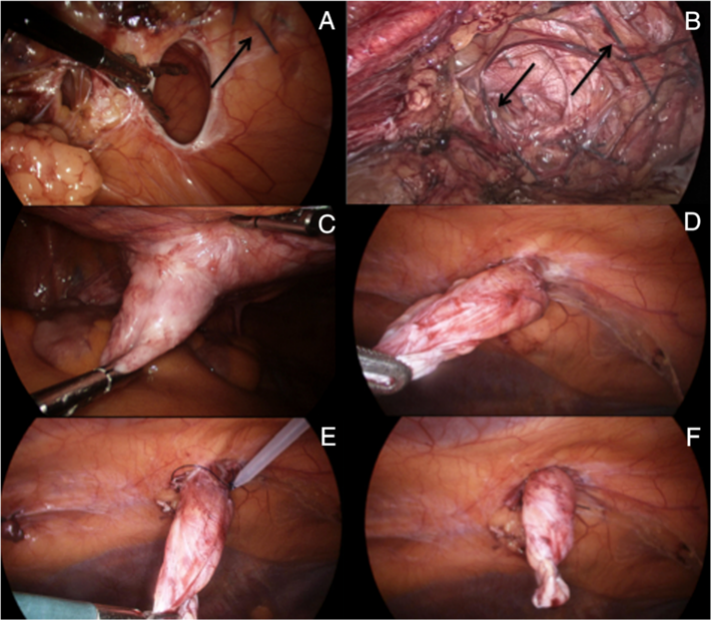
**a** + **b**) ‘Swiss-cheese’ incisional hernia (arrows showing previous suture material); **c** + **d**) Inversion of hernia sac; **e** + **f**) Plication of hernia sac with Endoloop ligature of PDS

Few closure methods have been proposed including the transabdominal ‘shoelacing’ technique as described by Orenstein et al. [18]. I favour similar approach but our preference is to use a blunt Endo Close^™^ Trocar Site Closure Device (Covidien, Mansfield, MA, USA) for placing each absorbable suture [1 polydioxanone (PDS® II, Ethicon Endo-Surgery, Inc,] through the fascial edges as loose figure-of-eight (Fig. 4). The number of sutures will depend on the size of the defect. Once all sutures have been positioned, they are pulled up together until maintaining the edges of the defect in juxtaposition and successively knotted (Fig. 5). In the presence of ‘Swiss-cheese’ incisional hernia where the defects are most of the time small and multiple (Fig. 6), I recommend simple closure of the hernia sacs using a pre-tied suture loop of PDS (Endoloop® Ligature, PDS® II, Ethicon Endo-Surgery, Cincinnati, OH, USA), as previously described for plication of the lax transversalis fascia during endoscopic TEP mesh repair of direct inguinal hernia defects [19].

If conventional IPOM technique is indicated, the hernia defect can be accurately measured either by placement of spinal needles through the abdominal wall, or using a small disposable ruler that can be easily placed intraperitoneally. The size of the defect can be also calculated directly on the skin, but bear in mind that due to the curvature of the abdominal wall external measurements are always overestimated. This difference increases with the size of the patient and is accentuated by the pneumoperitoneum, reason why from now on the intra-abdominal pressure is maintained at a maximum of 8 mmHg in order to reduce this magnification phenomenon.

As previously mentioned, the aim is to select a prosthesis that extends 3 to 5 cm beyond all edges of the defect, especially if the defect has not been primarily closed. There are several ‘anatomical’ circumstances where a smaller overlap is acceptable and sometimes necessary. For instance when fixing the mesh to the pubic tubercle, under the costal margin, or on the iliac crest. This will be discussed later.

**Fig. 7:**
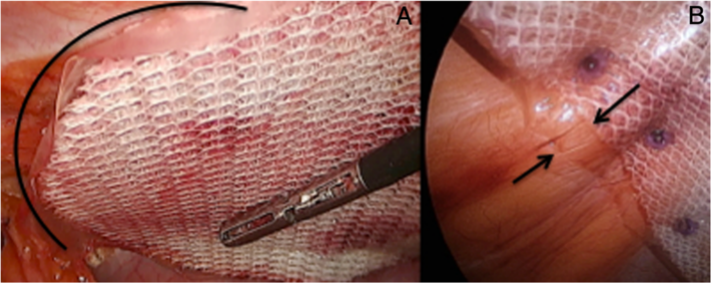
**a**) Hydrophilic polyester mesh easily positioned onto abdominal wall; **b**) Arrows showing absorbable collagen barrier being slightly wider than its polyester component

The choice of the mesh is a personal matter. The main rule is that the visceral side of the selected product must have anti-adhesive properties (titanium, collagen, ePTFE, cellulose) in order to reduce the risk of small bowel adhesions and potentially, delayed complications such as enteric fistula formation. The anti-adhesive barrier can be absorbable as the parietal peritoneum eventually remesothelializes within around one week [20]. Our preference is to use a large macroporous, three-dimensional (3D) scaffold and two-sided polyester mesh with an absorbable collagen barrier on the visceral side [Parietex^™^ Optimized Composite (PCOx) Mesh, Covidien, Mansfield, MA, USA]. This 3D design promotes rapid fibrous ingrowth rather than encapsulation, thus minimizing the risk of mesh ‘shrinkage’. Furthermore, the softness and hydrophilic properties of polyester allow an easier placement of the mesh onto the abdominal wall. Another particularity of this prosthesis is the fact that the absorbable collagen barrier is slightly wider than its polyester component (Fig. 7). Therefore if the edge of the mesh does curl after fixation (which is not uncommon) the risk of adhesion formation between the polyester material and the underlying viscus is greatly reduced. Another notable advantage with the PCOx mesh is that the softness offered by the polyester material permits a very easy insertion through the 12 mm port with minimal resistance, especially with bigger mesh sizes as compared to polypropylene or ePTFE based prostheses. Different sizes are available and the dimension of the mesh will be adapted to the abdominal wall defect. It is important to remember that in case of incisional hernia repair, the entire incision needs to be covered by the mesh and not just the hernia defect as failure to do so is recognized as a significant risk factor for recurrence [21].

Once the mesh size has been appropriately chosen and orientated its configuration is directly drawn with a sterile pen onto the abdominal Steri-Drape, surrounding the hernia defect. Of importance is that the mesh must be hydrated in a sterile saline solution before being inserted into the abdominal cavity. Dual method of mesh fixation with tackers and transfacial sutures is our preferred technique. On average six sutures of absorbable 2/0 polydioxanone (PDS® II, Ethicon Endo-Surgery, Inc., CA) are initially positioned along the periphery of the prosthesis before the mesh being rolled (collagen side on the inside) and delivered into the abdomen, via the 12 mmHg size port. The two ends of each of those sutures should have the same length in order to facilitate subsequent mesh fixation. One 2/0 PDS is long enough to place two sutures.

**Fig. 8:**
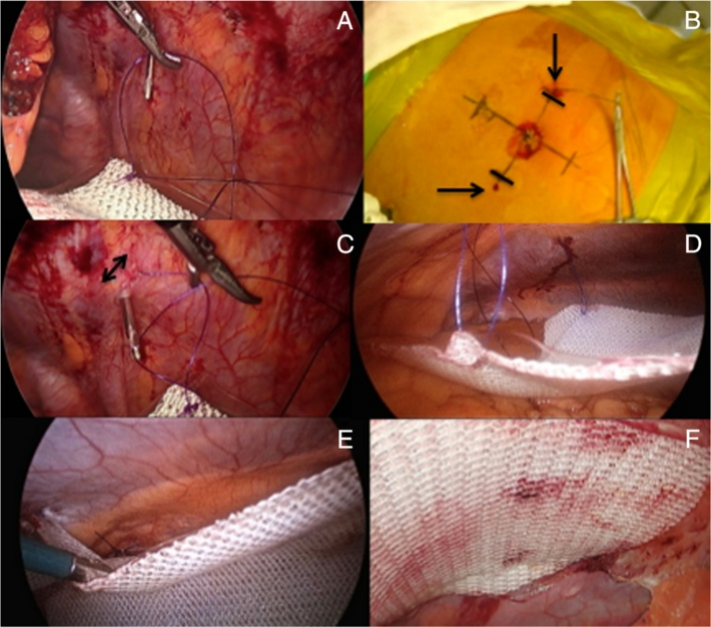
**a**) Each pair of pre-tied 2/0 PDS sutures separately retrieved with Endoclose device; **b**) Stab incisions (arrows) placed outside the (marks) mesh edges; **c** + **d**) Trans-abdominal sutures pulled out through different passageways; **e**) Good mesh overlap of previously closed hernia defect; F) mesh secured to posterior surface of abdominal wall

Wherever the sutures have been attached onto the mesh those positions are also measured and marked on the Steri-Drape, but around 2 cm away from the edges of the outlined mesh in order to reduce the risk of mesh folding, when subsequently tying the knots (Fig. 8b). Again, any distance calculated laparoscopically is always smaller than on the outside of the abdomen. Tiny stab incisions are made at the site of these marked positions. The PCOx mesh is then unrolled with laparoscopic graspers and orientated, with the collagen barrier facing the underlying viscus. Each end of the trans-abdominal sutures is pulled out from the abdominal cavity through different passageways with the help of an Endo Close^™^, but through the same stab incision (Fig. 8a, c, d). Both ends are then maintained under tension with crile forceps and the position of the mesh controlled before tying the knots one by one (Fig. 8e). At this stage, the mesh should appear centrally loose (sagging) but still lying up under tension against the abdominal wall (Fig. 8f). The knots remain tight in the subcutaneous tissues and the overlying skin released of any underlying attachment in order to reduce wound indentation.

**Fig. 9:**
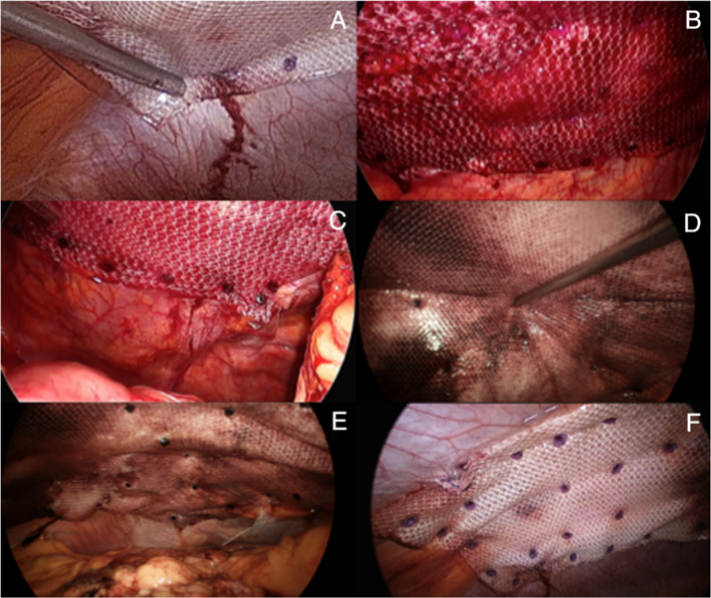
**a**-**d**) Mesh secured at 2cm intervals around periphery with absorbable tackers; **e**, **f**) Tackers applied more unevenly to maximize overall ‘contact’ between mesh and parietal peritoneum

Several laparoscopic fixation devices, either permanent or absorbable are currently available on the market. My preference today is to anchor the mesh with absorbable tackers (Sorbafix™, Bard Davol® Inc., Warwick, RI) as they seem, in my personal experience, to reduce the risk of developing chronic abdominal wall pain as compared to permanent one. Those tackers are fixed along the edge (outer layer) of the prosthesis 2 cm apart from each other (Fig. 9a-d). In case of a conventional IPOM repair, a ‘double crown’ technique is used meaning that an inner layer of fixation devices is also applied around the defect [22]. If the defect has been primarily closed (IPOM-Plus technique), then the tackers will be applied more unevenly onto the prosthesis in order to maximize the overall ‘contact’ between the mesh and the parietal peritoneum, thus reducing the prosthetic surface area that would be otherwise sagging from the abdominal wall, while at the same time most probably improving local tissue ingrowth (Fig. 9e, f). In order to obtain a better anchorage the abdominal wall is pressed down in opposite direction every time a tacker is applied, thus creating a counteraction.

#### Laparoscopic TransAbdominal PrePeritoneal (TAPP) technique

Currently, laparoscopic IPOM is the most frequently used technique, as it is relatively straightforward and requires reasonably short operating time. A concern is that following hernia repair adhesions may still develop in a significant number of patients and irrespective of the materials used [23], even though Chelala et al. [24] have reported a merely 11 % incidence of serosal adhesions formation with the polyester-based mesh Parietex^™^ Composite (precursor of the PCOx Mesh), in 85 redo surgeries after LRVIH. Nevertheless, ideally the prosthetic material should be placed in the extraperitoneal or retrorectus space as per the Rives-Stoppa principles [7, 8], but the laparoscopic TAPP approach is technically more challenging and will require longer operating time. In those situations I would recommend placement of an IDC at the beginning of the procedure. In the few cases where the mesh could be entirely secured in the extraperitoneal space, then a simple uncoated mesh would be sufficient (and cheaper) as there would be no potential risk of adhesion formation with the underlying viscus, separated by the peritoneum. The TAPP technique is mainly used in the lower abdomen when the hernia extends towards the symphysis pubis following a low midline laparotomy or Pfannenstiel incision post gynaecological surgery. Indeed, in these circumstances it is imperative to enter the extraperitoneal space, push back the bladder and expose the major vascular and nerve structures in the pelvis, otherwise the mesh cannot be properly and safely secured inferiroly.

**Fig. 10:**
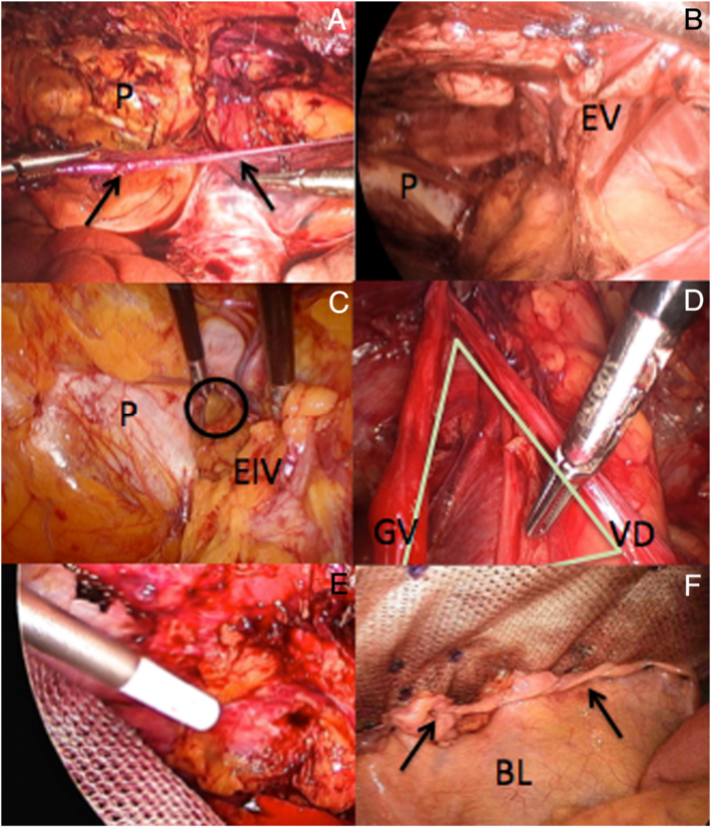
**a**) Parietal peritoneum (arrows) incised transversally (P: Pubic bone); **b**) Laparoscopic view of the extraperitoneal space (EV: Epigastric vessels); **c**) Reduced right femoral hernia (O) medial to the external iliac vein (EIV); **d**) Left ‘Triangle of Doom’ (VD: Vas deferens, GV: Gonadal vessels); **e**) Fibrin glue fixation of the infero-lateral aspects of the mesh; **f**) Closure parietal peritoneum (arrows) over distal part of the mesh (BL: Bladder)

The parietal peritoneum is incised transversally with hook diathermy and the dissection extended caudally towards the pelvis in the retropubic (Retzius) space (Fig. 10a). During that process, the epigastric vessels are identified, preserved and kept attached superiorly to the rectus abdominis muscles (Fig. 10b). Following those vascular structures distally allow us to identify other anatomical landmarks on both sides that will become progressively visible, namely the iliopubic tract, the medial aspect of the external iliac vein and femoral canal, and the pectineal (Cooper’s) ligament. When exposing the iliac veins, diathermy should be only used with extreme caution. Furthermore, in about one third of the cases an aberrant obturator vessel may be present and could lead to unexpected bleeding if not properly recognized*.* This vessel branch off from the inferior epigastric or less commonly external iliac vessels; crosses the superior pubic ramus and end up into the obturator foramen. Finally, in male the vas deferens runs medially and inferiorly, crossing over the external iliac vessels as it descends into the pelvis. By systematically exposing the femoral canal any associated femoral hernia (far more common in female patients), which would lie medial to the external iliac vein can be identified and reduced (Fig. 10c).

On some occasions it might be also necessary to extend our blunt dissection laterally towards the deep inguinal ring and posteriorly with exposure of part of the psoas muscle and the genito-femoral nerve that runs downwards on it. In those cases I recommend identification and reduction of any associated inguinal hernia, and in female patients to divide the round ligament (corresponds embryologically to the vas deferens). Once divided, the proximal end of the ligament may be secured on with an Endoloop of PDS if judged necessary as it may sometimes bleed. After completion of the dissection, the ‘triangle of doom’ and to a lesser degree the ‘triangle of pain’ will be exposed and it is very important to be familiar with those two anatomical danger zones before considering placement of the prosthetic mesh. The ‘triangle of doom’ is bordered medially by the vas deferens, laterally by the gonadal vessels and inferiorly by the reflected peritoneum, with its apex corresponding to the deep inguinal ring (Fig. 10d). It contains the external iliac vessels, the deep circumflex iliac vein as well as the genital branch of the genito-femoral nerve. The ‘triangle of pain’ lies laterally from this, delimited medially by the gonadal vessels, superiorly by the iliopubic tract and laterally by the reflected peritoneum. Within this triangle lies the femoral branch of the genito-femoral nerve, the lateral cutaneous femoral nerve of the thigh and more deeply the femoral nerve [20]. No sutures or stapling material can be used in this region for mesh fixation.

When performing a laparoscopic TAPP repair I also prefer to primarily close the defect if feasible, select a PCOx mesh and fix the upper part of the prosthesis with sutures and absorbable Sorbafix™, similar to the IPOM-Plus technique. Caudally, the medial part of the PCOx mesh is anchored onto the pubic symphysis, but both infero-lateral aspects of the mesh have to be fixed differently as it is unsafe to use either stapling or suturing devices in this region. In this situation and similar to our previously published TEP technique for inguinal hernia [25], I secure this part of the prosthetic mesh with 4mls of fibrin glue (TISSEEL [Fibrin Sealant], Baxter, Deerfield, Il, USA) uniformly sprayed (Fig. 10e). When adequately positioned the mesh should stretch out from the retropubic space, lean against the posterior aspect of the superior pubic ramus inferiorly, and lying infero-laterally over the psoas muscle. This way, the mesh will also reinforce the Hesselbach’s triangle and cover all the potential hernia sites in this region; namely direct, indirect, femoral and obturator. Once the PCOx mesh has been completely secured the previously incised parietal peritoneum is closed, thus partially covering the distal end of the prosthesis as shown on Fig. 10f.

Although quite uncommon, incisional hernia of the subxiphoid region may also occur following a median sternotomy, insertion of a chest tube in the mediastinum or simply after a laparoscopic procedure (such as cholecystectomy) where a 10mm working port has been placed in the epigastrium. The difficulty of the repair is mainly due to the close proximity of the rib cage and xiphoid process, but also the diaphragm, pericardium and pleural cavity. In this case, it is preferable to have the patient in a lithotomy and reverse Trendelenburg position, and perform part of the dissection from between the legs with the TV monitor located cephalad towards the right shoulder. The retroxiphoid space must be entered to allow a subfascial position and adequate mesh overlap. To do so, and following adequate dissection of the *ligamentum teres* of the liver and falciform ligament (Fig. 3b, c) I prefer the use of a harmonic scalpel. Indeed this device is safe and offers a sharp tissue dissection if deemed necessary, with better haemostasis. Furthermore, the potential risk of small bowel injury is almost inexistent as the anterior surface of the liver generally prevents intestinal incarceration [26]. Once the posterior lamina of the rectus sheath that inserts on the posterior side of the xiphoid process has been divided, subsequent development of the retrosternal space can be achieved by blunt dissection.

**Fig. 11:**
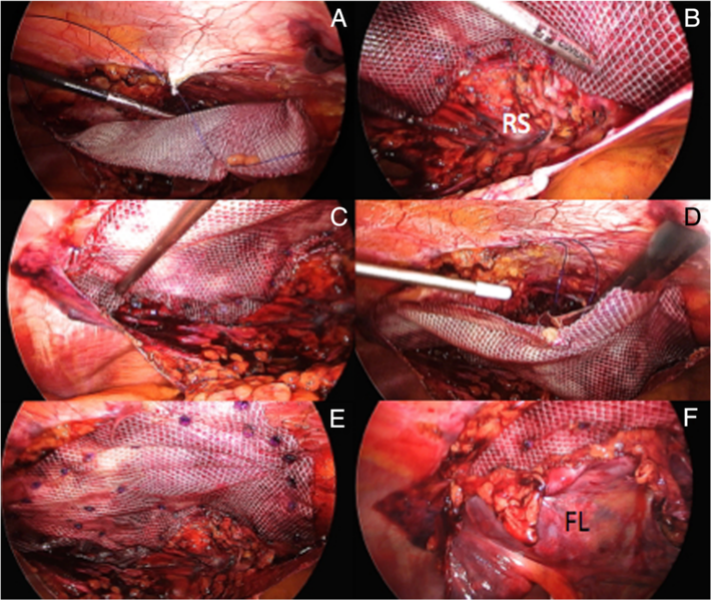
TAPP mesh repair of subxiphoid region. **a**) Transfacial sutures; **b, c, e**) Tackers fixation (RS: Retrosternal space); **d**) Fibrin glue fixation onto the antero-lateral chest wall; **f**) Falciform ligament (FL) reattached onto abdominal wall

Similar to the laparoscopic technique previously described, proper mesh fixation is achieved by a mix use of both transfacial sutures and tackers (Fig. 11a-c). As the most cephalad (proximal) portion of the prosthesis cannot be safely fixed with those devices, our preference is once again to glue the mesh onto the antero-lateral chest wall with fibrin sealant (Fig. 11d). Whenever possible, after completion of mesh fixation part of the previously dissected falciform ligament is reattached onto the abdominal wall thus covering part of the prosthesis (Fig. 11e, f).

#### Laparoscopic IPOM-plus and Totally Extraperitoneal (TEP) techniques for Spigelian hernia

**Fig. 12:**
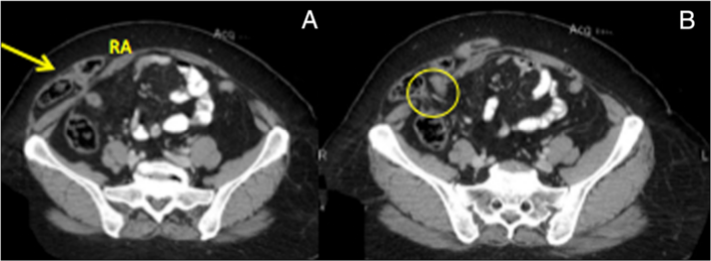
CT imaging of right Spigelian hernia. **a**) Hernia sac (arrow) containing loop of small bowel (RA: Right rectus abdominis); **b**) Abdominal wall defect (O)

**Fig. 13:**
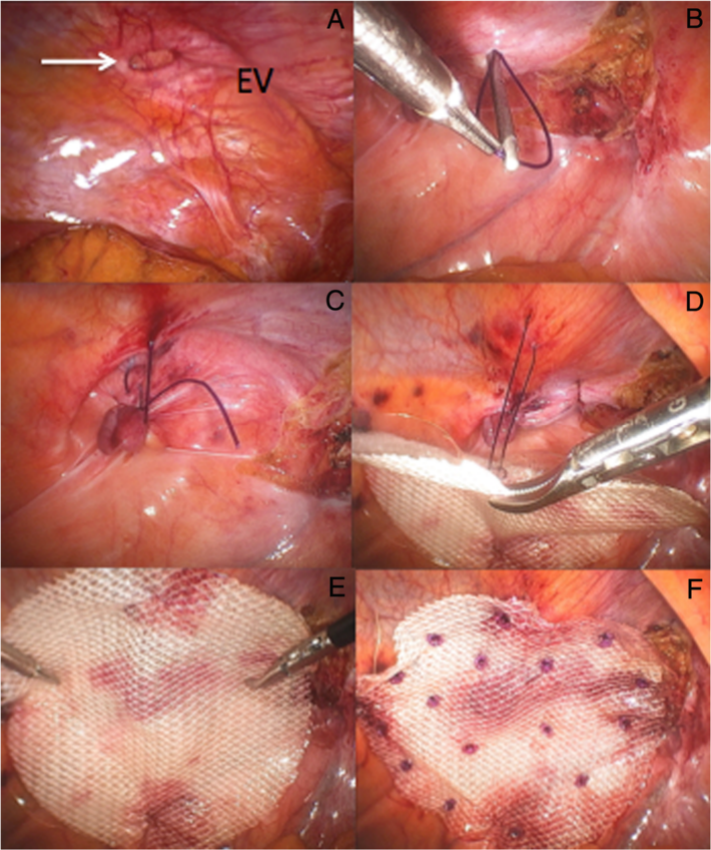
**a**) Left Spigelian defect (arrow; EV: Epigastric vessels); **b**, **c**) Primary closure defect with Endoclose; **d**, **e**) Mesh suspension with interrupted 2/0 PDS sutures; **f**) Polyester mesh secured with absorbable tackers

Spigelian hernia, or also called ‘spontaneous lateral ventral hernia’ arises at the level of the semilunar line (*Linea semilunaris*) just lateral to the rectus abdominis muscle and almost exclusively inferior to the umbilicus, at or below the level of the arcuate line that corresponds to the distal limit of the posterior rectus sheath. Most of the time the hernia is interparietal with no obvious mass on palpation and abdominal CT remains the best diagnostic tool (Fig. 12). Although quite uncommon, it is very useful to become familiar with different procedures for the repair of Spigelian hernia. Laparoscopic IPOM-Plus technique is undoubtedly the easier and quicker approach, applying the same principle as previously discussed. Figure 13 offers a good overview of the repair.

**Fig. 14:**
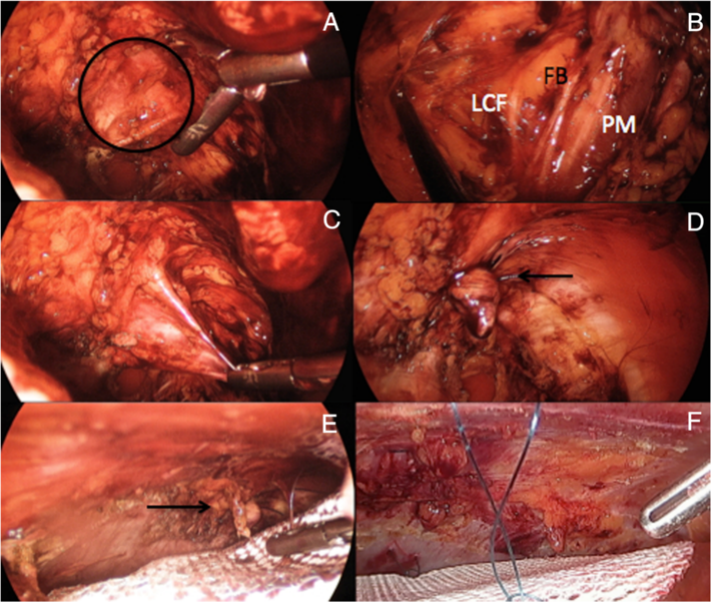
TEP repair of left Spigelian hernia. **a**) Abdominal wall defect; **b**) Left infero-lateral extraperitoneal dissection (LCF: Lateral cutaneous femoral nerve; FB: Femoral branch of the genito-femoral nerve; PM: Psoas muscle); **c, d**) Inversion and plication of weakened fascia with Endoloop PDS (arrow); **e**, **f**) Circular polyester mesh covering plicated (arrow) Spigelian fascia

Since the abdominal wall defect is often relatively small in size I prefer to perform a TEP repair, thus avoiding the unnecessary placement of a mesh into the abdominal cavity. The drawback is that the TEP technique is much harder to master as compared to IPOM. Similar to the TEP technique for inguinal hernia [25], the anterior rectus sheath is exposed and incised vertically via a lateral para-umbilical incision, making certain not to open the *linea alba*. The medial margin of the rectus abdominis muscle is retracted laterally allowing exposure of the posterior rectus sheath. A single use blunt-nosed 10mm lubricated dissection balloon trocar (Extra View^™^, Covidien, Mansfield, MA, USA) is then inserted in the preperitoneal space at an angle and advanced without force down to the symphysis pubis using a twisting motion and slight elevation of the trocars tip with the wrist, aiming for the centre of the pubic bone thus avoiding potential tearing of the epigastric vessels. The balloon trocar is inflated with air under direct vision thus progressively creating a preliminary extraperitoneal working space, and must remain inflated for about 3 min in order to achieve hemostasis by balloon tamponade. The balloon is then deflated and the trocar removed. A single use 10mm’s cannula (Blunt Tip trocars, Covidien) is then introduced into the preperitoneal space and secured as per manufacturer’s instruction. CO_2_ insufflation is kept at an ideal pressure of 8mm Hg. The camera is introduced through the paraumbilical port and the partly dissected preperitoneal space is visualized. Another two 5mm working ports are introduced within the preperitoneal space under direct vision on the opposite side of the Spigelian hernia and the extraperitoneal space carefully dissected laterally aiming at the anterior superior iliac spine (ASIS). Unless the Spigelian hernia is already completely reduced, the hernia sac is identified arising lateral to the rectus muscle and at the level of the arcuate line and completely reduced (Fig. 14a). The next step is to create a generous space around the defect sufficient enough to accommodate the prosthesis. The dissection often extends caudally towards the ‘triangle of pain’ where the femoral branch of the genito-femoral nerve and the lateral cutaneous femoral nerve lye (Fig. 14b). This way, proper deployment of the inferior border of the mesh can also be guaranteed. No sutures or stapling material can be used in this region for mesh fixation.

Quite often the defect is small and I therefore recommend primary closure with either Endoloops of PDS (Fig. 14c, d) or figure-of-eight of absorbable 1PDS as previously described (Fig. 4). Using a standard flat polyester or polypropylene mesh for hernia reinforcement would be sufficient as there is no risk of adhesion formation with underlying viscus, but I still prefer to use the round-shaped 9cm diameter PCOx mesh (occasionally the 12cm one) as it is soft, tends to spontaneously stick onto the abdominal cavity, pre-shaped and already containing two stay sutures (Fig. 14e, f). Furthermore, the mesh-to-defect ratio is such that the smaller round PCOx size is more than adequate. Finally, the mesh is preferentially secured with absorbable tacks, but in some occasions where the defect is very small fibrin glue alone may also be sufficient.

Whatever the technique used IPOM, IPOM-Plus, TAPP, or TEP, after satisfactory repair haemostasis is checked and preferentially no drain is used. The pneumoperitoneum (or extra-) is totally deflated under direct vision, local anesthetic is injected into the wounds and the skin closed with interrupted subcuticular 3/0 Caprosyn stitches. Steristrips and waterproof dressing are kept intact for five days. All patients are fitted with an abdominal binder just prior to being extubated and once fully awake they can resume a normal diet, and are able to walk freely if comfortable.

Intravenous antibiotic is generally given for the first 24 h and low molecular weight heparin ceased at the discretion of the operating surgeon. In some occasions, the patient might be discharged home (generally within two days) on extended VTE prophylaxis for few weeks. He is prescribed with simple analgesia and will need to remain on light duties with no straining or heavy lifting for a minimum of 3–4 weeks. Ideally I like to keep the binder tight 24/7 for two weeks and then daytime for another 4 weeks minimum. In our experience this significantly reduces the level of post-operative pain and also provides additional external support during the healing process. Follow up is usually organized at two weeks, six weeks and 3 months post-operatively. If deemed necessary, subsequent post-operative review is also scheduled until patients are symptom free and on some occasions a repeat abdominal CT-scan is organized at 6-month post-operative.
